# Anti-PD1 prolongs the response of PI3K and farnesyl transferase inhibition in *HRAS-* and *PIK3CA*-mutant head and neck cancers

**DOI:** 10.1016/j.neo.2025.101157

**Published:** 2025-03-20

**Authors:** Dinesh Babu Manikandan, Sankar Jagadeeshan, Sooraj Mathukkada, Raghda Abu Shareb, Manu Prasad, Liju Vijaya Steltar Belsamma, Divyasree Marripati, Noga Erez, Monica Wainer, Amit Geva, Danielle Raviv, Irit Allon, Luc GT Morris, Gloria H Su, Hai Wang, Ari J Rosenberg, Linda Kessler, Francis Burrows, Moshe Elkabets

**Affiliations:** a The Shraga Segal Department of Microbiology, Immunology, and Genetics, Ben-Gurion University of the Negev, Beer-Sheva, Israel; b Faculty of Health Sciences, Ben-Gurion University of the Negev, Beer-Sheva, Israel; c Department of Surgery, Memorial Sloan Kettering Cancer Center, New York, USA; d Institute of Pathology, Barzilai University Medical Center, Ashqelon, Israel; e Herbert Irving Comprehensive Cancer Center, Columbia University Medical Center, New York, NY, USA; f Department of Pathology, Columbia University Medical Center, New York, NY, USA; g Department of Otolaryngology/Head and Neck Surgery, Columbia University Medical Center, New York, NY, USA; h CAS Key Laboratory for Biomedical Effects of Nanomaterials & Nanosafety, CAS Center for Excellence in Nanoscience, National Center for Nanoscience and Technology, and University of Chinese Academy of Sciences, Beijing, China; i Department of Medicine, Section of Hematology and Oncology, University of Chicago, Chicago, IL, USA; j Kura Oncology, Inc., San Diego, CA, USA

**Keywords:** Head and neck cancer, HRAS, PI3K, Tipifarnib, Anti-PD1, Alpelisib

## Abstract

**Background:**

Tipifarnib, a farnesyl transferase inhibitor, has shown promising response in the treatment of *HRAS*-mutant HNSCC in the clinic, and in combination with a PI3K inhibitor in *PIK3CA*-mutant mouse models; however, the involvement of antitumor immunity in the efficacy of tipifarnib has not yet been investigated. This study aimed to evaluate the involvement of antitumor immunity in the efficacy of tipifarnib in *HRAS*- or *PIK3CA*-mutant HPV-positive and HPV-negative head and neck cancer murine models.

**Methods:**

To investigate the role of antitumor immunity, we compared the efficacy of tipifarnib in immune-intact C57BL/6 mice and immunodeficient NSG mice. Histopathological analyses were conducted to evaluate PD-L1 expression and the activation of key signaling pathways. Additionally, the synergistic potential of tipifarnib with the PI3Kα inhibitor alpelisib (BYL719) was assessed *in vitro* and *in vivo*. Immunohistochemical analysis was performed to examine the infiltration of CD8^+^*T* cells, and anti-PD1 treatment was tested to evaluate its potential to prolong progression-free survival.

**Results:**

In the HPV-positive *HRAS*-mutant HNSCC model, the antitumor efficacy of tipifarnib was primarily dependent on CD8^+^*T* cell activity, whereas in HPV-negative cancers, the contribution of antitumor immunity was less pronounced. Tipifarnib treatment upregulated PD-L1 expression, potentially inhibiting T cell antitumor activity and inducing hyperactivation of the AKT pathway, which mitigated MAPK inhibition and promoted cell proliferation. Blocking the PI3K pathway with alpelisib demonstrated synergistic antitumor effects in all models. The combination of tipifarnib and alpelisib exhibited greater efficacy in immune-intact mice than in immunodeficient mice, and was accompanied by increased CD8^+^*T* cell infiltration. Adding anti-PD1 treatment to the tipifarnib/alpelisib combination further prolonged progression-free survival in tumor-bearing mice.

**Conclusion:**

These findings underscore the critical role of antitumor immunity, particularly CD8^+^*T* cell activity, in the efficacy of tipifarnib alone and in combination with alpelisib. The triple combination of tipifarnib, alpelisib, and anti-PD1 treatment showed superior antitumor activity and extended survival in preclinical models, suggesting its potential as a therapeutic strategy for HNSCC patients with *HRAS*- and *PIK3CA*-mutation.

## Highlights

• Tipifarnib efficacy depends on CD8^+^ T cells in HPV-positive *HRAS*-mutant HNC model. In HPV-negative HNC, AKT activation and PD-L1 upregulation limit tipifarnib efficacy.
• Alpelisib enhances tipifarnib efficacy by blocking MAPK and AKT/mTOR signaling *in vitro*.
• Tipifarnib/alpelisib combination increases CD8^+^ T cell infiltration but upregulates PD-L1 expression *in vivo*.
• Triple combination of tipifarnib, alpelisib, and anti-PD1 significantly prolongs progression-free survival, by providing unprecedented antitumor efficacy.
• The study provides rationale for tipifarnib/alpelisib/anti-PD1 combination in *HRAS/PIK3CA*-mutant HNSCC patients.

## Graphical abstract

**Figure:**
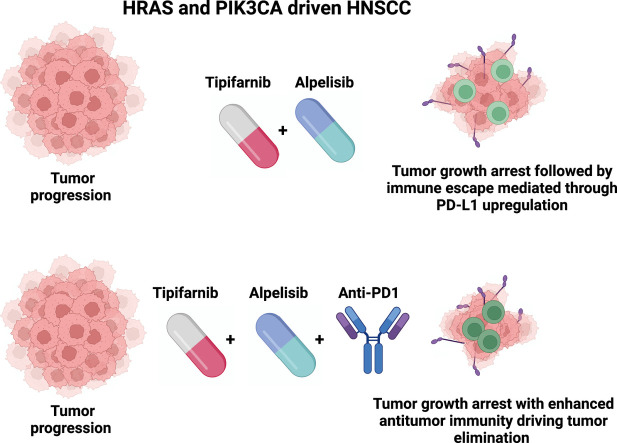
Effect of combination therapy of tipifarnib, alpelisib and anti-PD1 on *HRAS* and *PIK3CA* mutant HNSCC tumors.

## Introduction

Head and neck squamous cell carcinoma (HNSCC) is a highly heterogeneous disease that encompasses several histological subtypes and anatomical locations, each presenting distinct clinical behaviors and risk factors [1]. Genetic alterations in HNSCC contribute significantly to this diversity, with common mutations occurring in genes such as *TP53, CDKN2A, PIK3CA, HRAS* and alterations in RTK and MAPK signaling pathways [[2], [3], [4], [5]]. Recent advances in genomic profiling have identified actionable mutations [[6], [7], [8], [9]] that can be targeted using personalized therapies in HNSCC (EORTC-1559-HNCG, NCT03088059) [9]. Although the response to these monotargeted therapies is potent in a fraction of patients, there is a risk of innate or acquired resistance that can result in disease progression or relapse. Therefore, it is essential to develop therapeutic approaches to improve the care of HNSCC patients.

Alterations in the HRAS and PI3K pathways are frequent in HNSCC [10] and represent promising targets for therapy. *HRAS* mutations are found in approximately 7 % of HNSCC cases [11], and HRAS overexpression has been observed in up to 30 % of HNSCC cases [12], suggesting that some *HRAS* wild-type (WT) HNSCCs may exhibit a certain level of HRAS dependence. *PIK3CA* is the second oncogene in which gain-of-function mutations or *PIK3CA* amplification are present in approximately 30 % of patients with HNSCC [12]. This heterogeneity and the genetic landscape of HNSCCs support the potential for tailored treatment approaches to improve patient outcomes and optimize therapeutic strategies [13]. *HRAS* mutation/overexpression and *PIK3CA* mutations/amplifications account for up to 50 % of HNSCC cases [14]. Co-targeting of HRAS and PI3K has offered a promising therapeutic strategy, as simultaneous inhibition was shown to provide synergistic effects, enhance antitumor activity, and overcome drug resistance in animal models [12,15]. Preclinical studies [12,15,16] have suggested that combining the farnesyl transferase inhibitor (FTI) tipifarnib, which blocks HRAS activity, with alpelisib, a PI3Kα inhibitor, effectively suppresses tumor growth in genetically defined subsets of HNSCC, and a clinical trial is currently in progress (NCT04997902) [14]. In *PIK3CA*-mutant tumors, alpelisib activity is limited by the adaptive reactivation of mTORC1 signaling, which can be blocked by tipifarnib-mediated defarnesylation of the essential mTORC1 activator RHEB [12,14]. While preclinical studies have been performed in cell lines *in vitro* and patient-derived xenografts in immunodeficient mice, the involvement of antitumor immunity in this combination approach has never been studied.

By studying the response to tipifarnib in an immunocompetent model, we observed that the antitumor efficacy of tipifarnib depends more on CD8^+^ *T* cell activity in HPV-positive *HRAS*-mutant HNC, whereas in HPV-negative *HRAS*-mutant HNC, the hyperactivation of AKT and PD-L1 limits its efficacy. Dual treatment comprising anti-PI3K therapy (alpelisib) and tipifarnib was associated with CD8^+^ *T* cell infiltration, and supplementation of the tipifarnib/alpelisib combination with anti-programmed cell death protein-1 (anti-PD1) prolonged the progression-free survival of tumor-bearing mice.

## Materials and methods

### Cell culture

The murine *HRAS*-mutant cell line mEERL (mouse oropharyngeal epithelial cells expressing HPV16 E6/E7, HRAS, and luciferase) [17,18] was obtained from Dr. Paola D Vermeer at the Cancer Biology Research Center, Sanford Research (Sioux Falls, South Dakota) and maintained in E-media (Dulbecco's modified Eagle's medium (DMEM) containing 10 % fetal bovine serum, 22 % Ham's F12, 1 % penicillin/streptomycin, 25 μg/mL hydrocortisone, 5 μg/mL transferrin, 5 μg/mL insulin, 1.36 ng/mL tri-iodo-thyronine, and 5 ng/mL epithelial growth factor) as described previously [[17], [18]].

The FT1 cell line was developed in our laboratory from 4NQO-induced tongue cancer in female C57/BL6 mice. Briefly, fresh tongue tumor tissues were washed with HBSS or PBS, cut into small pieces with sterile scissors, and treated with an enzyme mixture of collagenase (10 mg/ml; catalog 17104019; Thermo Fisher Scientific), hyaluronidase (1 mg/ml; catalog H3506; MilliporeSigma), and DNase (200 U/ml; Thermo Fisher Scientific, catalog 18047019). Tissues were dissociated using a gentle MACS Dissociator. Cells were filtered through a 70-μM cell strainer, centrifuged (300 × g, 5 min), and cultured in DMEM (**Supplementary Table 1**). The cultures were stained for the epithelial markers, cytokeratin 14 and E-cadherin, to verify their epithelial origin. Genomic sequencing of 468 cancer-related genes using the MSK-IMPACT platform showed that the FT1 cell line harbored many mutations, including *HRAS* (G12E) and *Tp53* (G263*) (**Supplementary Table 1**). Murine PIK3CA mutant cell lines S24-658 (PIK3CA^+/H1047R^/p53^+/R172H^/k14cre^+^) and S26-117 (PIK3CA^H1047R^/k14cre^+^) [19] were kindly gifted by Prof. Gloria H. Su, Herbert Irving Comprehensive Cancer Center, Columbia University Irving Medical Center, New York.

The FT1 and HRAS^V12^ shp53 EpT (generated in our lab) [15,20], as well as S24-658 and S26-117 cell lines were maintained at 37°C in a humidified atmosphere at 5 % CO_2_, in DMEM supplemented with 1 % l-glutamine (200 mM), 100 units each of penicillin and streptomycin, and 10 % FBS. Cells were routinely tested for mycoplasma infection and treated with appropriate antibiotics, if needed (De-Plasma, TOKU-E, D022).

### IC_50_ assay

Cells were seeded in 96-well plates (5000 cells/well), treated with increasing concentrations of tipifarnib, and allowed to proliferate for 72 h. Each well received 50 μl of MTT (3-(4,5-dimethylthiazol-2-yl)−2,5-diphenyltetrazolium bromide) solution (5 mg/ml) and was incubated for 3 h. The 96-well plates were covered with aluminum foil and incubated at 37°C for 3 h. The purple formazan product was dissolved by adding 100 μl of DMSO to each well. The absorbance was measured at 570 nm using (BioTek Epoch spectrophotometer). The dose-response curve was plotted, and the IC_50_ values were calculated using the GraphPad® Prism 9 software.

### Colony formation assay

Cells were seeded in a 12-well plate (1000 cells per well) and treated the following day. After 24 h, cells were exposed to the indicated concentrations of tipifarnib (1 μm), alpelisib (2 μm) or a combination of alpelisib and tipifarnib for 72 h. The cells were then incubated in drug-free medium for 7–10 days to allow colony formation. The colonies were stained with 0.25 % crystal violet and counted based on three independent manual counts.

### Cell proliferation assay

Cells were seeded in 24-well plate (10 000–20 000 cells per well) and treated the following day. At the endpoint, the cells were stained with the MTT solution (5 mg/ml). Quantification was performed by dissolving MTT in DMSO and measuring the optical density at 570 nm using a spectrophotometer (Epoch Biotech).

### Determination of synergy

Cells were seeded in 96-well plates (3000 cells/well), treated with varying concentrations of the relevant drugs in combination, and allowed to proliferate for three days. Cells were then stained with MTT, and each well received 50 μl of MTT solution (5 mg/ml). The 96 well plates were covered with aluminum foil and incubated at 37°C for 3 h. The purple formazan product was dissolved by adding 100 μL DMSO to each well, and the absorbance was measured at 570 nm using a BioTek Epoch spectrophotometer (Winooski, Vermont, USA). For synergy assays, the proliferation of cells in the different treatment groups was determined as the percentage of control (DMSO-treated) cells, and the percentage of growth inhibition was calculated. ZIP Synergy scores were calculated using the SynergyFinder website [21].

### Western blotting

Cells were harvested and lysed using lysis buffer (50 mM HEPES, pH 7.5, 150 mM NaCl, 1 mM EDTA, 1 mM EGTA, 10 % glycerol, 1 % Triton X-100, 10 µM MgCl_2_) supplemented with phosphatase inhibitor (Biotool, B15001A/B) and protease inhibitor (Millipore Sigma, P2714-1BTL) cocktails and placed on ice for 30 min, followed by 3 min of sonication. The lysates were cleared by centrifugation (30 min, 14,000 rpm, 4°C). Supernatants were collected, and whole-cell lysates (25 µg) were separated by 10 % SDS–PAGE and blotted onto PVDF membranes (Bio-Rad Trans-Blot® Turbo™ transfer pack #1704157). Membranes were blocked for 1 h in blocking solution [5 % BSA (Amresco 0332-TAM) in Tris-buffered saline (TBS) with 0.1 % Tween] and then incubated with primary antibodies diluted in blocking solution. Mouse and rabbit horseradish peroxidase (HRP)-conjugated secondary antibodies were diluted in blocking solution. Protein-antibody complexes were detected using chemiluminescence [Westar Supernova, Cyanagen Cat. XLS3.0100), and Westar Nova 2.0 (Cyanagen Cat. XLS071.0250)], and images were captured using the Azure C300 Chemiluminescent Western Blot Imaging System (Azure Biosystems). Details of the antibodies used are presented in **Supplementary Table 2**.

### *In vivo* experiments

Mice were housed in air-filtered laminar flow cabinets with a 12-h light/dark cycle and supplied with food and water *ad libitum*. All animal experiments were carried out under the Institutional Animal Care and Use Committee (IACUC) of Ben-Gurion University of the Negev (BGU's IACUC) according to specified protocols to ensure animal welfare and reduce suffering. The animal ethical clearance protocol numbers used for the study were: IL.80-12-2015 (E), IL.29-05-2018 (E), IL.43-06-2019 (E), and IL.44-06-2019 (E). *In vivo* experiments were conducted using 6–8-week-old NSG mice (NOD.Cg-Prkdcscid Il2rgtm1Wjl/SzJ, Jackson Labs, Bar Harbor, ME, USA) and WT mice (Envigo, Huntingdon, UK, C57BL/6 J). The study was conducted in accordance with the regulations of the Association for Assessment and Accreditation of Laboratory Animal Care, ensuring the ethical treatment of the animals involved.

### *In vivo* models for drug efficacy studies

To generate tumor-bearing mice, cells (5 × 10^6^) were suspended in 100 μl of PBS and injected subcutaneously into the right and left flanks of 6-week-old male C57BL/6 J (WT) mice. For the orthotopic model, cells (5 × 10^6^) were suspended in 50 μl of PBS and injected into the lips of 6-week-old C57BL/6 J (WT) mice or 6-week-old NSG mice. Tumor-bearing mice were then randomized into groups of four–six mice, based on the tumor volume (between 150 and 200 mm^3^ or 30-50 mm^3^ for orthotopic tumors in the lips). For all *in vivo* experiments, treatment was administered orally on a daily basis, consisting of vehicle (5 % DMSO, 5 % Tween 80, 40 % PEG 300, and 50 % PBS), monotherapy with tipifarnib (at a dose of 60 mg/kg, administered twice daily), alpelisib (at a dose of 25 mg/kg, administered daily), or a combination of drugs as outlined. For efficacy experiments with tipifarnib and anti PD-1 antibody, 60 mg/kg tipifarnib was used twice daily, and anti PD-1 (rat anti-mouse PD-1, BE0146-25 -clone RMP1-14) and IgG (rat anti-mouse IgG2a, BE0089-25) from Bio X Cell were used at concentrations of 100 µg/mouse. Tumors were measured twice a week using a digital caliper, and tumor volume was calculated according to the formula V = (L × W × W) π/2, where V is the tumor volume, W is the tumor width, and L is the tumor length. Tumor volumes are plotted as mean ± SEM. At the end of the experiment, the animals were sacrificed and the tumors were collected. Comparison of response between WT and NSG was done using the Compare Groups of Growth Curves.

### CD8 and CD20 depletion

*In vivo* Plus™ anti-mouse CD8α (rat anti-mouse CD8α, BP0061-25 Clone 2.43), mouse anti-mouse CD20 (clone MB20-115, Mouse IgG2c, Bio X Cell) or IgG *In vivo* Plus ™ rat IgG2b isotype control, from Bio X Cell, were used for the CD8 and CD20 depletion experiment. The mice were administered 1 mg/mouse of anti-CD8 antibody or IgG intraperitoneally 2 days before tipifarnib treatment, and treatment was continued with 500 µg/mouse of anti-CD8 antibody or IgG every 5 days. For B cells depletion, mice were treated with a single dose of 100 μg of anti-mouse CD20.

### Immunohistochemistry

Tissues were fixed in 4 % paraformaldehyde (PFA) solution for a maximum of 24 h at room temperature, dehydrated, and embedded in paraffin. The tissue sections were deparaffinized with xylene. H_2_O_2_ (3 %) was used to block endogenous peroxidase activity for 20 min, after which the sections were rinsed in water for 5 min. Antigen retrieval was performed in citrate buffer (pH 6) at 99.99°C for 15 min. Sections were then blocked for 1 h at room temperature with blocking solution [phosphate buffered saline (PBS), 0.1 % Tween, 5 % bovine serum albumin (BSA]), followed by incubation with primary antibody (diluted in blocking solution) overnight at 4°C. An ABC kit (VECTASTAIN® Cat. VE-PK-6200) was used for color detection according to the manufacturer's protocol. Sections were counterstained with hematoxylin and mounted in mounting medium (Micromount, Leica Cat. 380-1730). All slides were digitalized using a Panoramic Scanner (3DHISTECH, Budapest, Hungary), and the analysis was performed using Qupath-0.2.3 software. The fields of tumor tissue analysis were chosen by a blinded investigator. Cells within the analysis field were detected using Qupath-0.2.3 software and were defined as positive or negative for DAB staining according to a threshold set by two independent blinded investigators. The cell detection criteria and thresholds were maintained between the slides. The specificity of the staining and analysis threshold was verified by comparison with a matched negative control tissue, which was incubated without a primary antibody, but was subjected to all secondary antibody development processes. Details of the antibodies used are presented in **Supplementary Table 2.**

### Statistical analysis

Statistical analysis was performed using GraphPad® Prism software (version 9), and the values are presented as the mean ± SEM. For comparisons between two groups, P values were calculated using an unpaired t-test. One-way ANOVA was performed using Tukey's multiple comparison test for experiments with more than two groups. P values of 0.05 (*), 0.01 (**), 0.001 (***), and 0.0001 (****) were considered statistically significant.

## Results

### Tipifarnib efficacy is enhanced by antitumor immunity

**Fig. 1:**
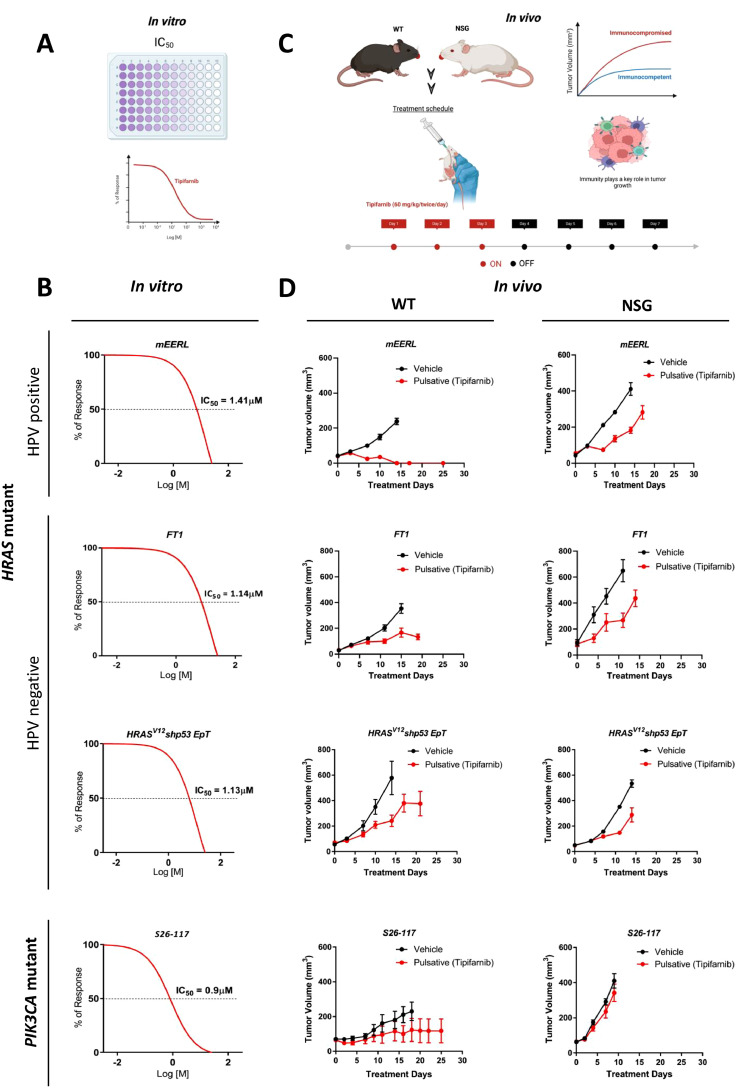
Tipifarnib efficacy is enhanced by antitumor immunity. (A) Schematic representation of the *in vitro* viability assay using tipifarnib (created using www.biorender.com). (**B**) Viability of HPV-positive *HRAS* mutant (mEERL), HPV-negative *HRAS*-mutant (FT1, HRAS^V12^ shp53 EpT), and *PIK3CA*-mutant (S26-117) cell lines treated with increasing doses of tipifarnib treatment for four days and the IC_50_ values are shown. (**C**) Schematic representation of the pulsative treatment regimen (tipifarnib 60 mg/kg by oral administration, twice daily for three days) and the comparison of WT vs*.* NSG mice (created using www.biorender.com). (**D**) Tumor volume of the mEERL, FT1, HRAS^V12^ shp53 EpT, and S26-117 syngeneic murine models in WT and NSG mice; 3  ×  10^6^ cells were injected orthotopically, mice were randomized into two groups. (n = 5) treated with vehicle or tipifarnib.

To explore whether the efficacy of tipifarnib is affected by host immunity, we used murine HNC cell lines, namely the HPV-positive *HRAS* mutant cell line – mEERL [17,18], two HPV-negative *HRAS*-mutant cell lines - HRAS^V12^ shp53 EpT [15,20] and FT1 (HRAS^G12E^p53^G263*^) (developed in our laboratory) and two HPV-negative *PIK3CA*-mutant cell lines - S24-658 (PIK3CA^+/H1047R^/p53^+/R172H^/k14cre^+^) and S26-117 (PIK3CA^H1047R^/k14cre^+^) [19]. First, we evaluated the susceptibility of these cell lines to tipifarnib *in vitro* (Fig. 1A). The half inhibitory concentrations (IC_50_) of tipifarnib for mEERL, HRAS^V12^ shp53 EpT, FT1, S26-117, and S24-658 were found to be 1.41 µM, 1.14 µM, 1.13 µM, 0.9 µM and 2.09 µM respectively (Fig. 1B**, Supplementary Figure 1A**).

Pulsatile treatment regimens utilizing tipifarnib demonstrated enhanced suppression of tumor growth in clinical settings [22]. To elucidate the role of the immune system in the response to pulsatile administration of tipifarnib, we injected mEERL, FT1, HRAS^V12^ shp53 EpT, and S26-117 into the lips of immunocompromised NOD/SCID/IL2rγ^null^ (NSG) and immunocompetent WT mice. Subsequently, we compared tumor growth between cohorts treated with tipifarnib (60 mg/kg) on a three-day-on, four-day-off schedule (Fig. 1C) and those administered vehicle alone. In NSG mice, tipifarnib treatment resulted in the slow progression of mEERL, FT1, and HRAS^V12^ shp53 EpT tumors, whereas rapid progression was observed in S26-117 tumors. In syngeneic immune-intact WT mice, tipifarnib treatment caused complete regression of mEERL tumors, whereas tipifarnib delayed tumor progression in HPV-negative HRAS-mutated tumors (Fig. 1D). Comparison on the response between WT and NSG using the compare groups of growth curves shows significant differences in all *HRAS-*mutant tumors. These *in vivo* results suggest that host antitumor immunity may enhance the efficacy of tipifarnib, thereby preventing tumor progression.

### Tipifarnib treatment led to CD8^+^*T* cell-dependent tumor elimination in HPV-positive *HRAS*-mutant tumor, whereas in HPV-negative *HRAS*-mutant tumor, hyperactivation of AKT pathway and overexpression of PD-L1 were associated with progression to tipifarnib

**Fig. 2:**
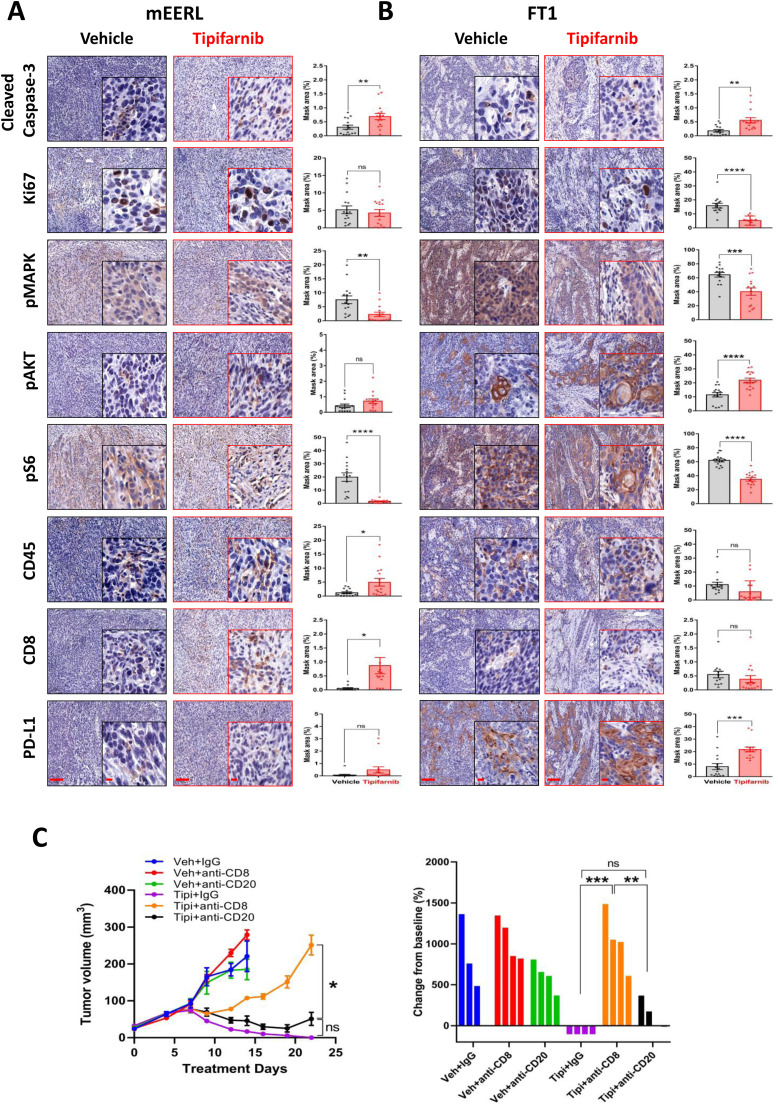
Tipifarnib induces AKT activation in tumor cells and promotes immune escape *via* PD-L1 upregulation in HPV-negative *HRAS* mutant HNSCC, whereas tipifarnib efficacy depends on CD8^+^*T* cell infiltration in HPV-negative *HRAS* mutant HNSCC. (A) Representative immunohistochemical staining images showing the expression of cleaved caspase-3, Ki67, pMAPK, pAKT, pS6, CD45, CD8, and PD-L1 in mEERL tumors treated with vehicle or tipifarnib (60 mg/kg) for three days (scale bars: 100 μm; insets 20 μm). Quantification of positive cells (n = 3 tumors and n = 15 analysis fields). Error bars indicate SEM. Statistical significance was calculated using one-way ANOVA (*p < 0.05, **p < 0.01, ***p < 0.001, ****p < 0.0001); ns denotes not significant). (B) IHC images showing the expression of cleaved caspace-3, Ki67, pMAPK, pAKT, pS6, CD45, CD8, and PD-L1 in FT1 tumors treated with vehicle or tipifarnib (60 mg/kg) for three days (scale bars: 100 μm; insets 20 μm). Quantification of positive cells (n = 3 tumors and *n* = 15 analysis fields). Error bars indicate SEM. Statistical significance was calculated using a one-way analysis of variance (ANOVA; **p < 0.01, ***p < 0.001, ****p < 0.0001; ns denotes not significant). (C) Growth of mEERL tumors in WT mice: 3 × 10⁶ cells were injected orthotopically, and mice were randomized into six treatment arms (*n* = 6) with tipifarnib, with or without depletion of CD8^+^*T* cells and CD20^+^*B* cells. Right: Fold change in volumes of mEERL tumors treated with tipifarnib with or without depletion of CD8^+^*T* cells and CD20^+^*B* cells.

To further understand the differential response of HNC tumors to tipifarnib, we compared the pathological responses of mEERL and FT1 tumors before and after 3 days of treatment with tipifarnib. Immunohistochemical (IHC) analysis after 3 days of treatment revealed a notable increase in CD45^+^ and CD8^+^ *T*-cell infiltration in mEERL tumors compared to that in vehicle-treated controls (Fig. 2A). Conversely, FT1 tumors exhibited only an insignificant minimal elevation in these immune cell populations (Fig. 2B), suggesting that reduced CD8^+^ *T* cell infiltration may underlie the limited efficacy of tipifarnib in these tumors. Additionally, tipifarnib reduced the phosphorylation of MAPK and S6 in the mEERL tumors (Fig. 2A). In FT1 tumors, although MAPK and S6 phosphorylation decreased, AKT S473 phosphorylation significantly increased, potentially driving resistance (Fig. 2B). Due to the known role of AKT pathway in regulating PD-L1 expression [23,24], we investigated PD-L1 as a possible negative regulator of CD8^+^ *T* cell activity. PD-L1, which is known to dampen T cell activation and promotes T cell exhaustion [[25], [26], [27]], was significantly upregulated following tipifarnib treatment, as confirmed by IHC analysis. Based on this upregulation of PD-L1, we hypothesized that combining tipifarnib with an immune checkpoint inhibitor targeting PD-1 would mitigate immune suppression and enhance clearance in HPV-negative tumors. To test this hypothesis, a four-arm study was conducted in which tumor-bearing mice were treated with either IgG or anti-PD1, followed by vehicle or tipifarnib (60 mg/kg). Surprisingly, the tipifarnib/anti-PD1 combination did not improve progression-free survival in FT1 tumors, as tumor growth patterns were similar to those observed in the tipifarnib/IgG group (**Supplementary Figure 2A**). These results suggest that PD-L1 inhibition alone is insufficient to overcome resistance in FT1 tumors.

Given the established roles of CD8^+^ *T* and B cells in predicting better prognosis in HPV-positive HNSCC [[28], [29], [30]], we further explored their involvement in modulating tipifarnib efficacy. The mEERL tumor-bearing WT mice were divided into six treatment groups: vehicle + IgG, tipifarnib + IgG, vehicle + anti-CD8, vehicle + anti-CD20, tipifarnib + anti-CD20, and tipifarnib + anti-CD8. Depletion of CD8^+^ and CD20^+^ cells in the tumors was confirmed by IHC (**Supplementary Figure 2B**). Depletion of CD8^+^ *T* cells markedly accelerated tumor progression compared to IgG controls, and the antitumor effects of tipifarnib were significantly reduced in the absence of CD8^+^ *T* cells. All mice treated with tipifarnib + anti-CD8 developed tumors >150 mm³ within 20 days. Conversely, mice treated with tipifarnib + anti-CD20 exhibited delayed tumor progression, with none exceeding 100 mm³ at the same time point. Furthermore, tumor progression began earlier (15–16 days) in the tipifarnib + anti-CD8 group than in the tipifarnib + anti-CD20 group (20 days; Fig. 2C). These results highlight the essential function of CD8^+^ *T* cells in inhibiting mEERL tumor growth and boosting the effectiveness of tipifarnib, whereas CD20^+^ *B* cells play a secondary but complementary role.

### Inhibition of PI3K signaling by alpelisib improves tipifarnib efficacy *in vitro* by blocking MAPK and AKT/mTOR signaling

**Fig. 3:**
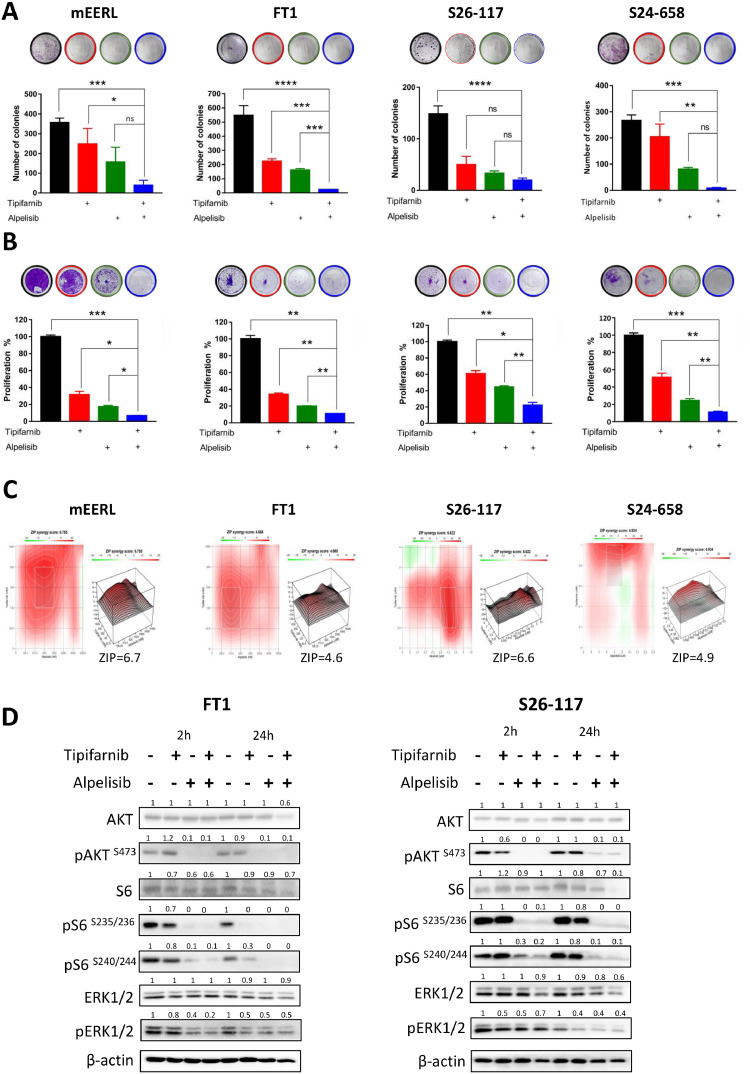
Enhanced efficacy of tipifarnib/alpelisib combination in both *HRAS* and *PIK3CA* mutant cells *in vitro*. (A) Colony formation assay with tipifarnib (1 μm), alpelisib (2 μm), and tipifarnib/alpelisib treatment in mEERL, FT1, S26-117, and S24-658 cell lines. (**B**) Four days cell proliferation assay was performed with tipifarnib (1μM), alpelisib (2 μm), and tipifarnib/alpelisib treatment in mEERL, FT1, S26-117, and S24-658 cell lines. (**C**) Synergy score and heat map calculated using SynergyFinder+ for the combination of tipifarnib and alpelisib in mEERL, FT1, S26-117, and S24-658 HNSCC cell lines. (**D**) Western blot analysis of the indicated proteins after 2 and 24 h of tipifarnib (1 μm), alpelisib (2 μm), and combination (tipifarnib/alpelisib) treatment in FT1 and S26-117 cell lines. Numbers indicate the fold-change in protein levels normalized to βactin. Data represent representative experiments from three independent experiments. Statistical significance was calculated using one-way ANOVA (*P < 0.05, **P < 0.01, ***P < 0.001, ****P < 0.0001).

Given that the PI3K/AKT pathway was activated in patient samples and HRAS-mutant cell lines [12,15], as well as in our murine model systems [15,19], we hypothesized that blocking the activated PI3K/AKT signaling using the p110α inhibitor alpelisib would enhance the efficacy of tipifarnib. To this end, we first examined the superior antitumor activity of tipifarnib/alpelisib using a colony formation assay (Fig. 3A) and found that the tipifarnib/alpelisib combination significantly inhibited the colony formation efficacy of the cells. The 2D proliferation assay results further supported the superiority of this combination over a single treatment (Fig. 3B). The antitumor interaction between the two inhibitors, tipifarnib and alpelisib, on tumor cells was further studied, and the synergistic scores for mEERL, FT1, S26-117, and S24-658 were 6.755, 4.668, 6.622, and 4.934, respectively (Fig. 3C). To examine the on-target effects, we profiled the signaling pathways using western blot analysis (Fig. 3D). Cell lysates were examined at 2 h and 24 h after treatment with tipifarnib, alpelisib, or a combination of the two drugs. Alpelisib treatment prevented tipifarnib-induced AKT hyperphosphorylation in FT1 cells (Fig. 3D). Combined exposure of cells to alpelisib and tipifarnib resulted in significant suppression of the MAPK, AKT, and mTOR signaling pathways, thereby reducing cell proliferation and survival. Taken together, these results further support the hypothesis that the PI3K blockade enhances the efficacy of tipifarnib.

### PI3K blockage enhance tipifarnib efficacy in both HRAS and PI3K mutant HNC

**Fig. 4:**
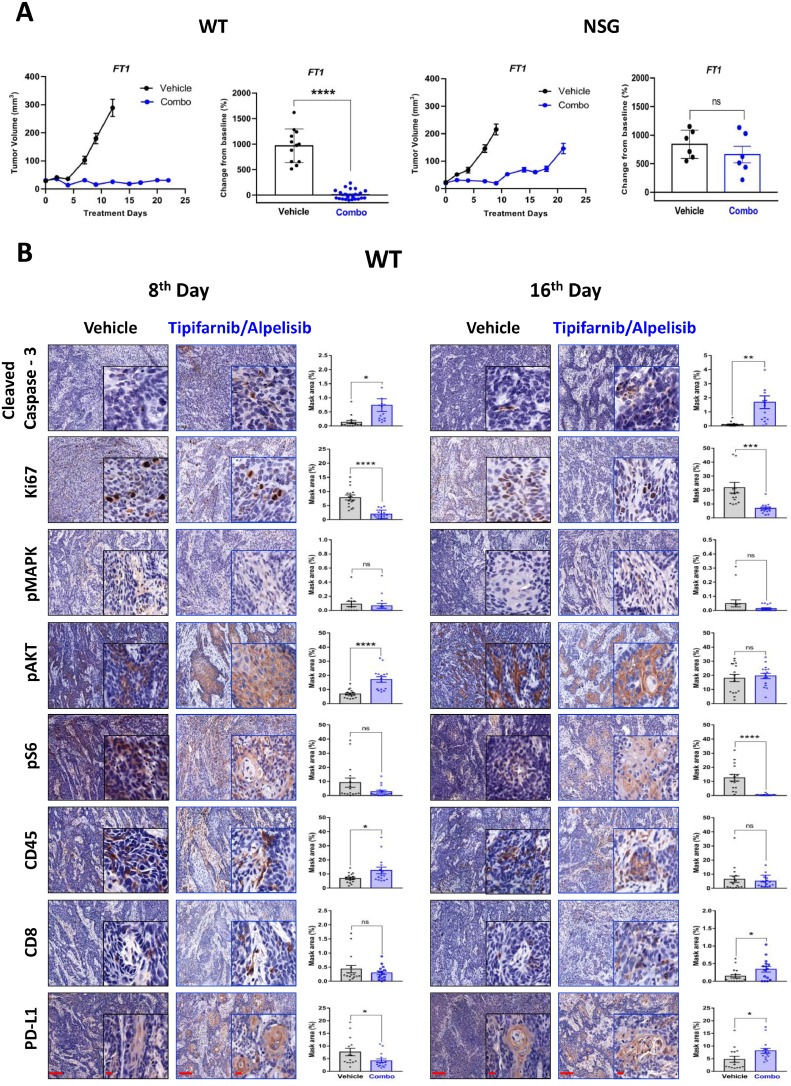
Tipifarnib/alpelisib displayed improved efficacy in HRAS and PIK3CA mutant in vivo models. (A) Tumor volume of FT1 syngeneic murine models of WT and NSG mice. 5  ×  10^6^ cells were injected subcutaneously, and mice were randomized into two groups. (WT, n = 14) and (NSG, *n* = 6) and treated with vehicle or tipifarnib/alpelisib [alpelisib (25 mg/kg daily once) and tipifarnib (60 mg/kg, twice daily]. (**B**) Representative immunohistochemical staining images of FT1 tumors harvested from WT mice at two different time points - 8^th^ and 16^th^ day, showing the expression of cleaved caspase-3, Ki67, pMAPK, pAKT, pS6, CD45, CD8, and PD-L1 in FT1 tumors treated with vehicle or tipifarnib/alpelisib (scale bars: 100 μm; insets: 20 μm). Error bars indicate SEM. Statistical significance was calculated using one-way ANOVA (*p < 0.05, **p < 0.01, ***p < 0.001, ****p < 0.0001; ns denotes not significant).

To investigate the role of antitumor immunity in the efficacy of tipifarnib/alpelisib combination, we compared its outcomes in WT and NSG mice. For this, 5 × 10⁶ FT1 cells were injected into the lips of both the mouse models. Treatment was initiated once the tumors reached 3–4 mm in diameter or 30–50 mm^3^ in volume. Combination therapy demonstrated distinct effects in both models. In NSG mice, tipifarnib/alpelisib initially exhibited strong antitumor activity until the 8^th^ day (Fig. 4A). However, by the 16^th^ day, the tumors had begun to progress, indicating the development of resistance. In contrast, WT mice showed disease stabilization during the same period, with no signs of tumor elimination or progression, suggesting that antitumor immunity effectively controlled tumor growth. Immunohistochemical analysis of tumor samples from WT mice treated with the combination revealed marked infiltration of CD8^+^ *T* cells, along with an increase in PD-L1 expression, particularly by the 16^th^ day (Fig. 4B). These findings highlight that while alpelisib enhances the efficacy of tipifarnib by reducing tumor proliferation and promoting CD8^+^ *T* cell-mediated antitumor activity, the upregulation of PD-L1 serves as a limiting factor, preventing complete tumor eradication. Taken together, these results underscore the dual impact of combination therapy: robust initial efficacy followed by immune-mediated stabilization in WT mice, and immune-independent resistance in NSG mice.

### Anti-PD1 treatment enhances the efficacy of the tipifarnib/alpelisib combination in mice

**Fig. 5:**
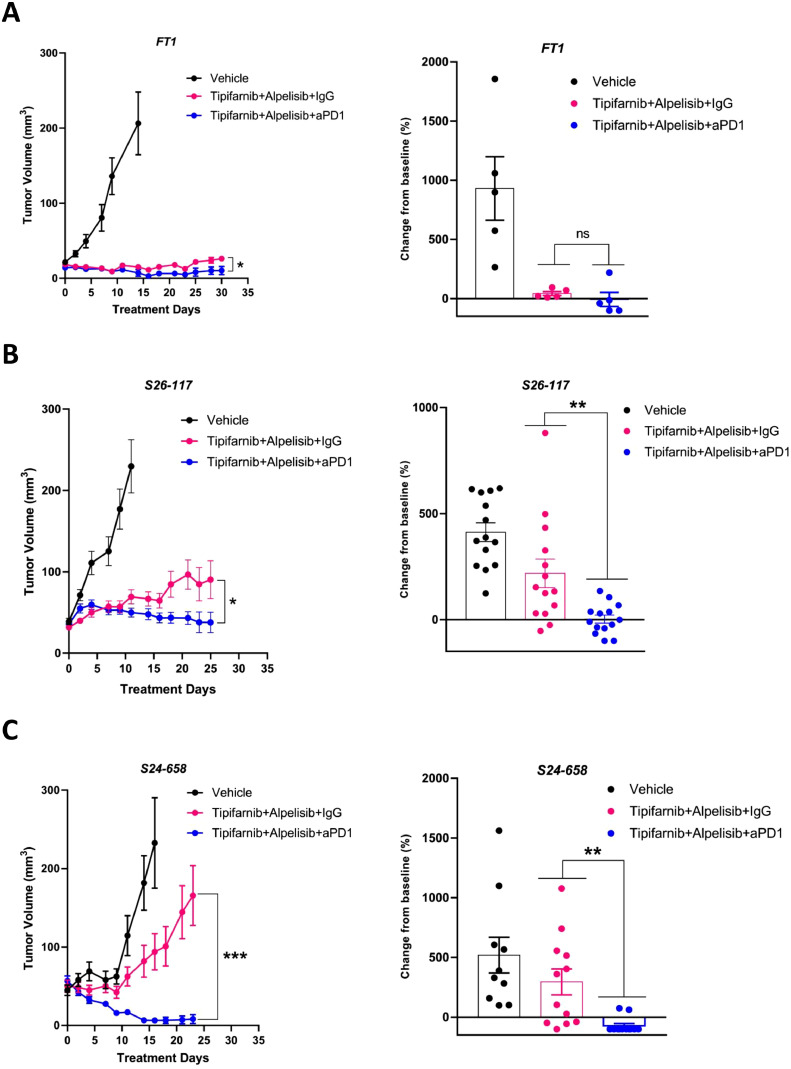
A triple combination of tipifarnib, alpelisib, and anti-PD1 promoted tumor regression in HPV-negative *HRAS-* or *PIK3CA-*mutant tumors. Tumor volumes of (A) FT1 syngeneic murine models in C57BL/6 J (WT) (n = 15, 5  ×  10^6^ cells were injected orthotopically into the lip), (B) S26-117, and (C) S24-658 syngeneic murine models in C57BL/6 J (WT) (n = 18, 5  ×  10^6^ cells were injected subcutaneously), and mice were randomized into three groups. and treated with vehicle, tipifarnib/alpelisib + IgG, or tipifarnib/alpelisib + anti-PD1 (*n* = 5-6). IgG and anti-PD1 were administered *via* I.P injection once every five days. Right: Fold changes in tumor volumes of FT1, S26-117, and S24-658 tumors treated with vehicle, tipifarnib/alpelisib + IgG, or tipifarnib/alpelisib + anti-PD1. Error bars indicate SEM. Statistical significance was calculated using one-way ANOVA (*p < 0.05, **p < 0.01, ***p < 0.001, ****p < 0.0001; ns denotes not significant).

To investigate whether combining an immune checkpoint inhibitor targeting PD-1 with tipifarnib/alpelisib enhanced antitumor immunity, improved tumor clearance, and prolonged response, we conducted experiments in WT mice using three tumor models: *HRAS*-mutant FT1 tumors, and *PIK3CA*-mutant S24-658 and S26-117 tumors. FT1 cells were injected orthotopically into the lip, whereas S24-658 and S26-117 cells were injected subcutaneously into the flank. Once the tumors reached a volume of 30–50 mm³, the mice were randomized into three treatment groups: tipifarnib/alpelisib + IgG, tipifarnib/alpelisib + anti-PD1, or vehicle controls. Among *HRAS*-mutant FT1 tumors, all mice (5/5) treated with tipifarnib/alpelisib + IgG exhibited tumor progression within 30 days, whereas 4/5 mice treated with tipifarnib/alpelisib + anti-PD1 achieved tumor elimination (Fig. 5A). For *PIK3CA*-mutant S24-658 and S26-117 tumors, the tipifarnib/alpelisib + anti-PD1 regimen also demonstrated superior efficacy (Fig. 5B-C). Tumors in all mice treated with tipifarnib/alpelisib + IgG progressed within 15 days, whereas those in mice treated with tipifarnib/alpelisib + anti-PD1 regressed after 15 days. Notably, S24-658 tumors showed more pronounced regression compared to S26-117 tumors under this combination therapy. Across all three syngeneic models, irrespective of *HRAS* or *PIK3CA* mutational status, the combination of tipifarnib/alpelisib with anti-PD1 significantly enhanced antitumor activity. These findings demonstrate the therapeutic potential of integrating anti-PD1 with tipifarnib/alpelisib combination.

**Fig. 6:**
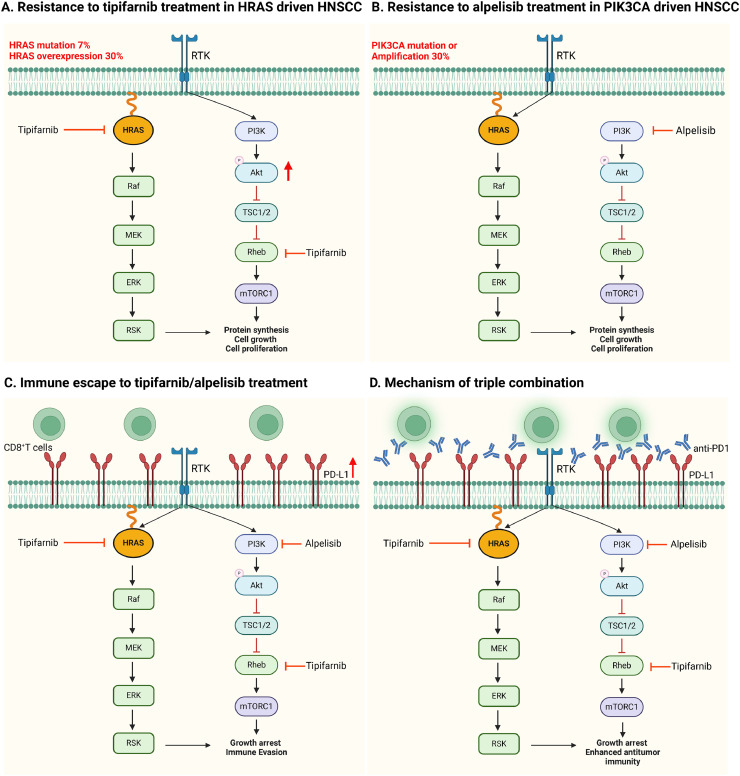
Diagrammatic illustration of the intrinsic and extrinsic responses of tumors with *HRAS/PIK3CA* mutations to various treatments: tipifarnib, alpelisib, the combination of tipifarnib and alpelisib, and triple therapy with tipifarnib, alpelisib, and anti-PD1. (A) Resistance to tipifarnib treatment in HRAS-driven HNSCC. Tipifarnib monotherapy in *HRAS*-mutant tumors resulted in AKT overactivation due to a compensatory mechanism involving the PI3K-AKT-mTOR signaling cascade, leading to tumor proliferation and growth. (B) Resistance to alpelisib treatment in PIK3CA-driven HNSCC. In tumors with *PIK3CA* mutations, treatment with alplelisib triggers hyperactivation of the RAS-MAPK pathway, serving as a compensatory mechanism to counteract the inhibition of the PI3K pathway, ultimately promoting tumor survival. (C) Immune escape following tipifarnib/alpelisib treatment. The combined administration of tipifarnib and alpelisib effectively inhibited the compensatory pathway activation. However, this dual treatment also created an immunosuppressive environment by increasing PD-L1 expression. Consequently, the activity of infiltrating CD8^+^*T* cells is impaired, resulting in immune evasion and tumor progression. (D) Mechanism of the tipifarnib/alpelisib/anti-PD1 triple combination: the synergistic use of tipifarnib, alpelisib, and anti-PD1 effectively inhibited the compensatory resistance pathway and PD-L1, thus enhancing antitumor immunity. This was achieved by boosting the activity of infiltrating CD8^+^*T* cells, which resulted in delayed tumor growth and subsequent tumor elimination (Created using www.biorender.com).

In summary, our findings confirm and extend our previous observations that treatment with tipifarnib leads to increased phosphorylation of AKT, representing an intrinsic mechanism of resistance driven by compensatory activation of the PI3K-AKT pathway upon RAS-MAPK inhibition [12]. Conversely, alpelisib treatment induces activation of the RAS-MAPK pathway, resulting in accelerated tumor progression and resistance. Notably, the combined inhibition of FTase with tipifarnib and PI3K with alpelisib effectively suppressed tumor growth; however, tumor relapse was observed due to the emergence of an immunosuppressive tumor microenvironment mediated by PD-L1 upregulation, which impairs CD8^+^ *T* cell function. Strikingly, the addition of anti-PD1 therapy to the tipifarnib/alpelisib combination elicited complete and durable regression of *HRAS*- and *PIK3CA*-mutant tumors, highlighting a compelling therapeutic strategy (Fig. 6).

## Discussion

Targeted therapies against HRAS and PI3K pathway components show promise for improving outcomes in HNSCC [4,31,32]. The farnesyl transferase (FT) inhibitor tipifarnib has shown promising results in treating patients with recurrent and metastatic HNSCC harboring *HRAS* mutations for whom treatment options are scarce [33]. With advances in our understanding of cancer signaling pathways, targeted therapies have the potential to improve the clinical outcomes in HNSCC patients [31,32,34]. Specifically, combining PI3K inhibitors such as alpelisib with complementary targeted agents like FTIs may offer an effective approach to overcome resistance mechanisms such as adaptive mTOR reactivation and improve outcomes [12,35]. Since preclinical studies have been conducted in cell lines *in vitro* and patient-derived xenografts in immunodeficient murine models, the extent of antitumor efficacy has not been thoroughly examined. This study investigated the role of antitumor immunity in the efficacy of a tipifarnib/alpelisib combination using *HRAS* and *PIK3CA* mutant HPV-positive and HPV-negative syngeneic models of head and neck cancer (HNC).

Patients with HPV-positive and HPV-negative HNSCC exhibited significant differences in treatment response, etiology, and mutational profiles. HPV-positive HNSCCs generally have a more favorable prognosis and better overall survival rates compared to HPV-negative tumors [36,37]. However, the underlying reasons for this are not fully clear and are likely due to a combination of factors, including enhanced immune responses against viral proteins and a more favorable tumor immune microenvironment. The presence of viral antigens in HPV-positive tumors may contribute to better immunogenicity, making these tumors more responsive to targeted therapies and potentially explaining their generally better prognosis compared to HPV-negative tumors [[38], [39], [40]]. In our study, by comparing the efficacy of tipifarnib in immunocompetent and immunodeficient mice, we observed that in the HPV-positive *HRAS*-mutant HNC model, the antitumor efficacy of tipifarnib was dependent on CD8^+^ *T* cell activity. Conversely, in HPV-negative cancers, the contribution of antitumor immunity was less pronounced. It is well known that tumors with and without HPV exhibit notable distinctions in their immune environments, with HPV-positive tumors typically demonstrating stronger immune responses [38,39,41]. Recent studies examining the immune microenvironment of HNSCC based on HPV status have revealed that HPV-positive HNSCC shows higher levels of B cells, plasma cells, CD8^+^ cytotoxic T cells, and CD4^+^ effector T cells. Conversely, these tumors have lower numbers of macrophages and mast cells than their HPV-negative counterparts [38,39]. Furthermore, it has been hypothesized that the heightened immune response observed in HPV-positive tumors correlates with improved clinical outcomes and increased patient survival [39]. Consistent with clinical observations of enhanced therapeutic responsiveness in HPV-positive HNSCC, the mEERL HPV-positive *HRAS* mutant mouse model displayed increased CD8^+^ *T* cell infiltration into tumors, resulting in tumor regression and eventual eradication when treated with tipifarnib. In a clinical trial investigating tipifarnib treatment in recurrent and metastatic HNSCC (NCT02383927), four HPV-positive and nine HPV-negative HNSCC patients were included, all of whom demonstrated an improved response to tipifarnib [22,42]. Interestingly, both *HRAS* and *PIK3CA* mutations are found in HPV-positive and HPV-negative tumors, but *PIK3CA* aberrations are enriched in HPV-positive tumors [37,43,44]. Our research was limited by the lack of a suitable syngeneic model for HPV-positive *PIK3CA*-mutant HNC, which prevented us from assessing the efficacy of tipifarnib in HPV-positive HNSCC with *PIK3CA* mutations.

Farnesyl transferase inhibitors (FTIs), such as tipifarnib, have shown potential for modulating the tumor immune microenvironment [[45], [46], [47], [48], [49], [50]]. A study by Liu et al. revealed that tipifarnib reduced the formation of tumor-associated macrophage precursors by modulating immature myeloid cells in the bone marrow [51]. Tipifarnib also inhibited LPS-induced responses in THP-1 leukemia cells by suppressing chemokines (MCP-1 and MCP-2), cytokines (IL-1β, IL-6, and IFN-β), signaling molecules (MyD88 and STAT-1), proteases (MMP-9), and receptors (urokinase receptor). In primary human peripheral blood mononuclear cells, tipifarnib inhibited TNF-α, IL-6, MCP-1, and IL-1β expression in a dose-dependent manner without cytotoxicity. *In vivo*, it reduced the production of these proinflammatory mediators in a murine model of LPS-induced inflammation. However, it did not affect LPS-induced IL-8 production. Mechanistically, tipifarnib partially inhibits p38 phosphorylation, blocks IκB-α degradation, and prevents nuclear translocation of p65 [46]. DeGeorge et al. showed that tipifarnib also inhibits NF-κB-mediated chemokine production, including CCL2 and CXCL1 [48]. These findings highlight the potential of tipifarnib in modulating immune responses, reducing inflammation, and influencing T cell function, making it a candidate for tumor immunotherapy. In our study, we observed infiltration of CD8^+^ *T* cells upon tipifarnib administration, independent of the HPV status. It is established that tipifarnib inhibits Th1 differentiation through the reduction of T-bet induction and modification of histone acetylation of the *IFN-γ* gene [45]. Furthermore, tipifarnib downregulated CXCL12 secretion, a critical factor for T cell homing and the maintenance of immune cell progenitors [52]. In HPV-negative HNC, despite the increased infiltration of CD8^+^ *T* cells, tipifarnib demonstrated limited efficacy, in part due to the upregulation of PD-L1. However, the addition of anti-PD1 in combination with tipifarnib did not enhance treatment efficacy (**Supplementary Figure 2A**), indicating that PD-L1 upregulation was not the sole factor contributing to the reduced efficacy. This finding suggests the potential involvement of additional factors that may either facilitate or confer resistance to tipifarnib.

The RAS-MAPK and PI3K/AKT signaling pathways collaboratively modulate mTORC1 and other effectors, thereby regulating cellular survival, metabolism, proliferation, and metastasis. Tipifarnib treatment upregulated AKT phosphorylation in HPV-negative *HRAS*-mutant models, consistent with previous studies indicating compensatory activation of the PI3K-AKT pathway [15]. Tipifarnib induces an increase in AKT activity, potentially reducing cancer cell sensitivity to these inhibitors. Compensatory PI3K/AKT pathway activation affects the susceptibility of cancer cells to FTI. Thus, the interaction between these oncogenic pathways may influence cancer sensitivity to targeted therapies such as FTIs. The activation of AKT following tipifarnib treatment may play a role in immune escape. For instance, it is well known that AKT activation can lead to upregulation of PD-L1 [24,53] and facilitate immune escape by regulating the secretion of immune suppressive factors [[54], [55], [56]]. The observed upregulation of PD-L1 in tissue samples after tipifarnib treatment and reduced treatment efficacy might be due to this feedback activation, as well as the immunosuppressive environment induced by the PI3K-AKT pathway. Hence, this finding suggests a potential pathway for combination therapy.

Evidence suggests that RAS and PI3K contribute to resistance to inhibitors of the alternative pathway [12,15,16,57,58]. Recent studies have shown that tipifarnib enhances the efficacy of alpelisib in HNSCC by suppressing both the HRAS-MAPK and PI3K-AKT-mTOR pathways [12,15]. This dual inhibition could help to counteract the feedback activation of these two major pathways, which are known to drive resistance to both FT and PI3K inhibitors. Using syngeneic models, we showed that the addition of alpelisib inhibited reactivation of the PI3K-AKT pathway by tipifarnib, and this combination enhanced the treatment efficacy of tipifarnib in *HRAS*-mutant HNSCC. An earlier investigation using preclinical models revealed that prolonged administration of tipifarnib/alpelisib ultimately resulted in tumor progression, owing to the development of resistance [15]. We also observed PD-L1 upregulation in the tissue samples from the combination therapy group, indicating a potential resistance mechanism. This observation led us to consider that enhancing antitumor immunity while tumors are still responsive to targeted therapy might extend progression-free survival. Through HNSCC preclinical syngeneic models, we demonstrated the efficacy of combining tipifarnib, alpelisib, and anti-PD1, providing a mechanistic rationale for advancing this combination to clinical trials. Our findings suggest that this approach could not only improve the effectiveness of tipifarnib/alpelisib but also broaden the potential of other approved or developing immune checkpoint inhibitors. Such advancements could significantly benefit recurrent and metastatic HNSCC patients.

Although our study provided compelling evidence supporting the therapeutic potential of the tipifarnib/alpelisib/anti-PD1 triple combination, it is essential to acknowledge certain limitations. First, our assumption that PD1 plays a critical role in CD8^+^ *T* cells in response to combination treatment is based on histological analyses that quantified cell numbers, but not their activation status. Further functional studies are required to confirm the activation and effector functions of these T cells. Secondly, the mechanism underlying the observed substantial infiltration of T cells induced by the tipifarnib/alpelisib combination remains unclear. Although it is plausible that this combination may reduce the secretion of immunosuppressive factors, this hypothesis warrants detailed investigation to elucidate the exact mediators involved. Thirdly, although our findings provide strong translational relevance for the triple combination therapy, we did not delineate the molecular mechanisms underlying the effects of tipifarnib/alpelisib/anti-PD1 treatment. Further studies are needed to explore the signaling pathways and molecular interactions that contribute to this synergy. Lastly, we observed upregulation of PD-L1 expression following treatment with tipifarnib/alpelisib; however, this has yet to be confirmed clinically. These limitations highlight areas for future research to deepen our understanding of the mechanisms driving the therapeutic effects of this combination and to further refine its clinical application.

## Conclusion

In conclusion, this study highlights the potential for a valuable role of antitumor immunity to enhance the efficacy of tipifarnib in the treatment of *HRAS-* and *PIK3CA*-mutant HNSCC. The research revealed that the effectiveness of tipifarnib is enhanced by CD8^+^ *T* cell activity, particularly in a single HRAS-mutant HPV-positive HNC model. However, in HPV-negative cancers, the contribution of antitumor immunity was less pronounced due to the hyperactivation of AKT and upregulation of PD-L1, which limited the efficacy of tipifarnib. The study demonstrated that combining tipifarnib with the PI3Kα inhibitor alpelisib resulted in synergistic antitumor activity across all tested models. This dual treatment approach showed superior efficacy in immune-intact mice compared to that in immunodeficient mice, further emphasizing the importance of the immune system in the treatment response. Importantly, the addition of anti-PD1 therapy to the tipifarnib/alpelisib combination prolonged progression-free survival in tumor-bearing mice. This triple combination of tipifarnib, alpelisib, and PD1 blockade shows promise as a potential treatment strategy for patients with *HRAS-* and *PIK3CA*-mutant HNSCC. These findings underscore the complex interplay between targeted therapies and the immune system during cancer treatment. Our findings also highlight the potential of combining immunotherapies to enhance treatment efficacy and improve patient outcome. Future clinical trials investigating this triple combination therapy in patients with HNSCC with specific genetic alterations are warranted to translate these preclinical findings into clinical practice.

## CRediT authorship contribution statement

**Dinesh Babu Manikandan:** Conceptualization, Data curation, Formal analysis, Investigation, Methodology, Writing – original draft, Writing – review & editing. **Sankar Jagadeeshan:** Conceptualization, Data curation, Formal analysis, Investigation, Methodology, Writing – original draft, Writing – review & editing. **Sooraj Mathukkada:** Conceptualization, Data curation, Formal analysis, Investigation, Methodology, Validation. **Raghda Abu Shareb:** Data curation, Investigation. **Manu Prasad:** Data curation, Investigation, Methodology. **Liju Vijaya Steltar Belsamma:** Data curation, Investigation, Methodology. **Divyasree Marripati:** Investigation. **Noga Erez:** Investigation. **Monica Wainer:** Investigation. **Amit Geva:** Investigation. **Danielle Raviv:** Investigation. **Irit Allon:** Data curation, Formal analysis, Investigation, Resources. **Luc GT Morris:** Formal analysis, Investigation, Resources. **Gloria H Su:** Formal analysis, Investigation, Resources. **Hai Wang:** Formal analysis, Investigation, Resources. **Ari J Rosenberg:** Formal analysis, Investigation, Resources. **Linda Kessler:** Investigation, Resources. **Francis Burrows:** Investigation, Resources. **Moshe Elkabets:** Conceptualization, Data curation, Formal analysis, Funding acquisition, Investigation, Methodology, Project administration, Resources, Software, Supervision, Validation, Visualization, Writing – original draft.

## Declaration of competing interest

The authors declare the following financial interests/personal relationships which may be considered as potential competing interests:Ben Gurion University of the Negev (investigator Prof. Moshe Elkabets) received a grant from Kura Oncology, Inc. Linda Kessler and Francis Burrows declare employment and stock or stock options with and travel expenses from Kura Oncology, Inc. This work was prepared while Gloria Su was employed at Columbia University Irving Medical Center. The opinions expressed in this article are the author's own and do not reflect the view of the National Institutes of Health, the Department of Health and Human Services, or the United States government. The remaining authors declare that they have no known competing financial interests or personal relationships that could influence the work reported in this study.

## Appendix Supplementary materials

Image, application 1

Image, application 2
