# Supplementary material for: Anti-PD1 prolongs the response of PI3K and farnesyl transferase inhibition in HRAS- and PIK3CA-mutant head and neck cancers
Source: Neoplasia. 2025 Mar 20;63:101157. doi: 10.1016/j.neo.2025.101157 (PMC11978339; doi:10.1016/j.neo.2025.101157)
Supplement: Supplementary file 1 [file mmc1.pptx]

## Slide 1
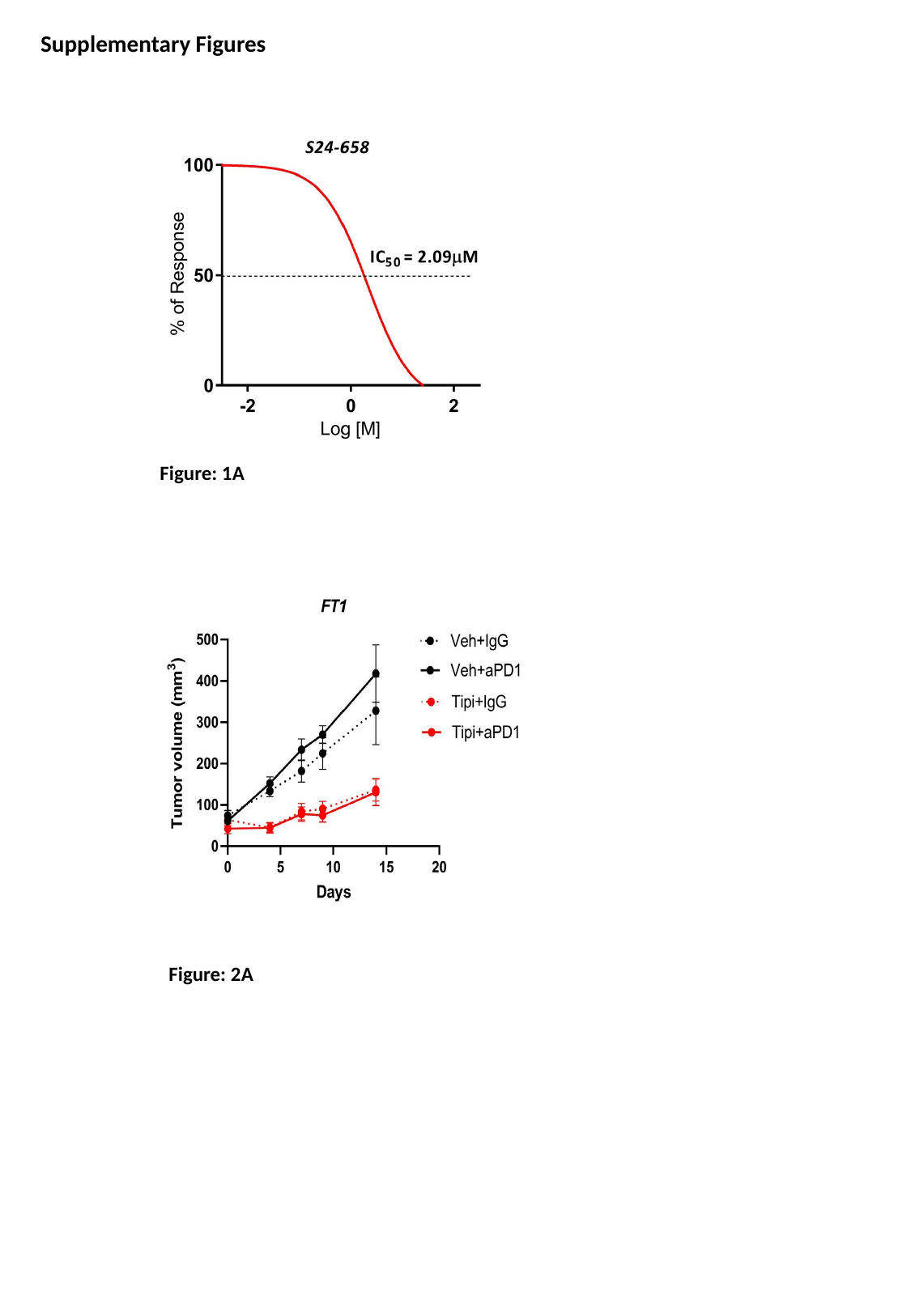

Supplementary Figures
Figure: 1A
Figure: 2A

## Slide 2
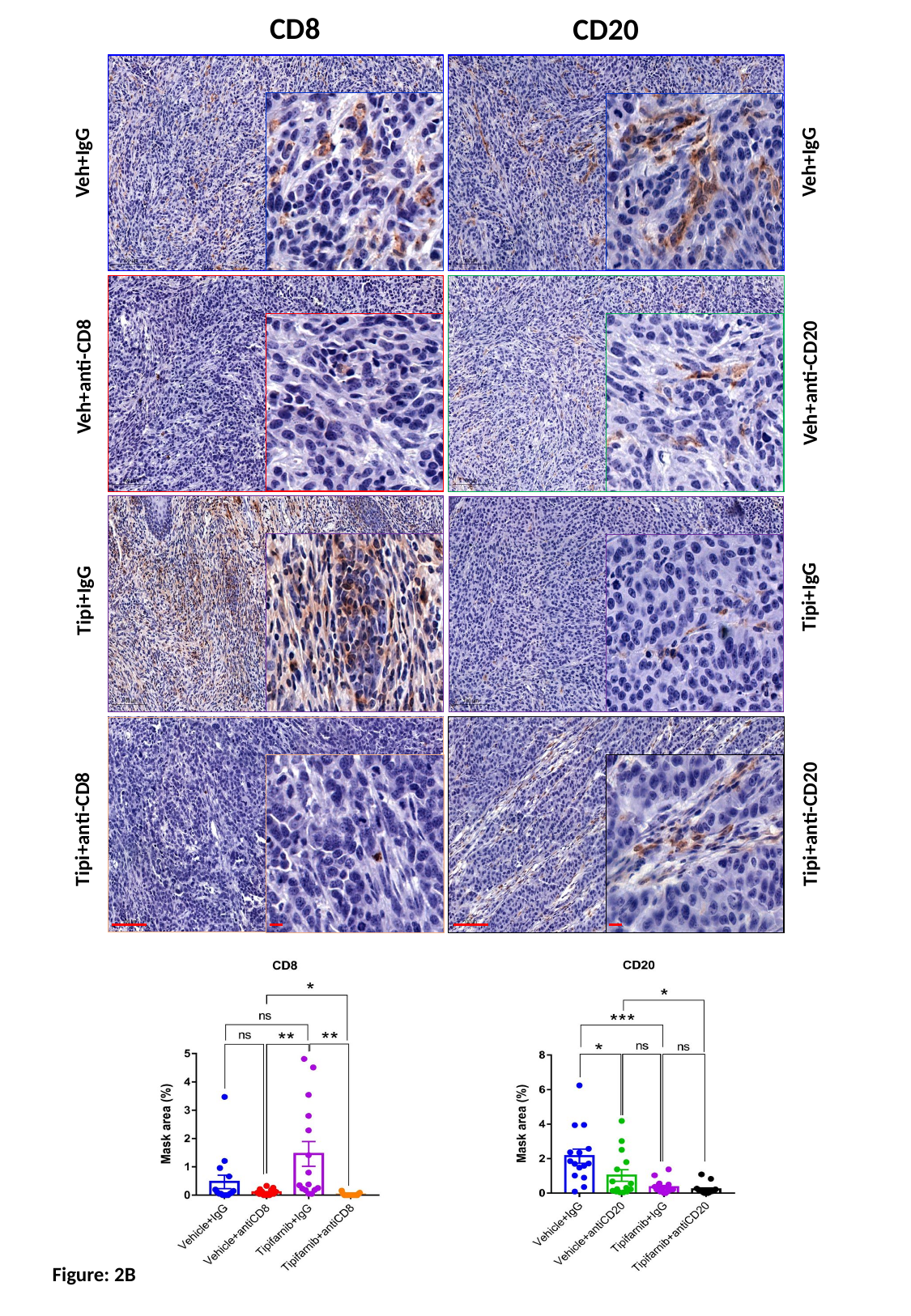

CD8
CD20
Veh+IgG
Veh+IgG
Veh+anti-CD8
Veh+anti-CD20
Tipi+IgG
Tipi+IgG
Tipi+anti-CD20
Tipi+anti-CD8
Figure: 2B
