# Supplementary material for: Anti-PD1 prolongs the response of PI3K and farnesyl transferase inhibition in HRAS- and PIK3CA-mutant head and neck cancers
Source: Neoplasia. 2025 Mar 20;63:101157. doi: 10.1016/j.neo.2025.101157 (PMC11978339; doi:10.1016/j.neo.2025.101157)
Supplement: Supplementary file 2 [file mmc2.docx]

**Supplementary Material**

**Anti-PD1 prolongs the response of PI3K and farnesyl transferase inhibition in *HRAS* and *PIK3CA* mutant head and neck cancers**

**Supplementary Figure Legends:**

**Figure 1A**: Viability of the HPV-negative PIK3CA mutant (S24-658) cell line treated with increasing doses of tipifarnib for four days and IC_50_ values are shown.

**Figure 2**: (**A**) Tumor volumes of FT1 in WT mice treated with IgG, anti PD-1, tipifarnib, or a combination of IgG or anti PD-1 and tipifarnib. (**B**) IHC images showing the expression of CD8 and CD20 in mEERL tumors treated with vehicle + IgG, vehicle + anti-CD8 or anti-CD20, tipifarnib (60 mg/kg) +IgG, tipifarnib + anti-CD8, or anti-CD20 (scale bars: 100 μm; insets 20 μm). Quantification of positive cells (n = 3 tumors and n = 15 analysis fields). Error bars indicate SEM. Statistical significance was calculated using one-way ANOVA (**p < 0.01, ***p < 0.001, ****p < 0.0001; ns denotes not significant).

**Schematic representation of the generation, culture and mutational analysis of FT1 cells. (**Created using www.biorender.com**)**


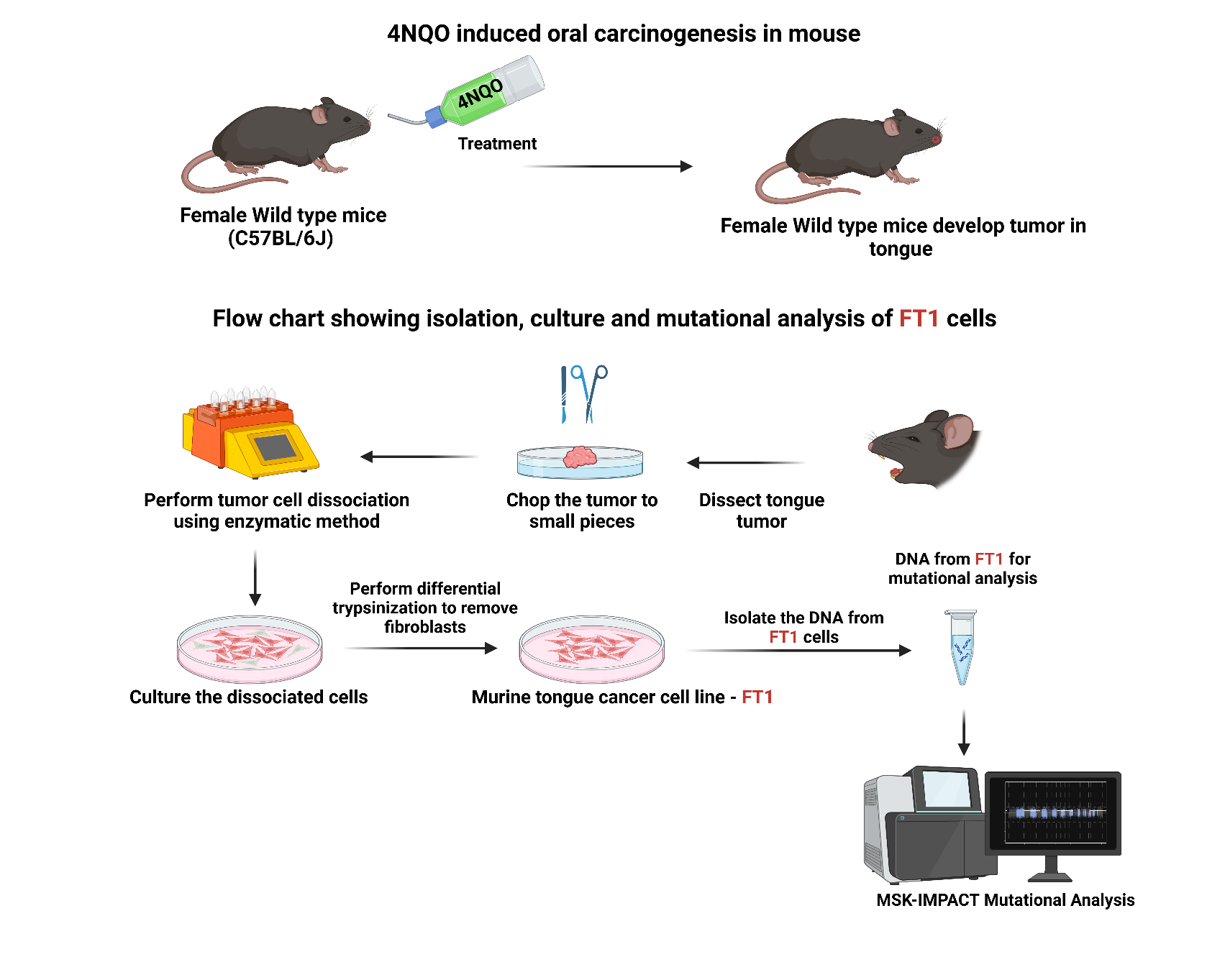


**Supplementary Table 1**

**Table 1: Mutational analysis of FT1 cells**

| Hugo_Symbol | HGVSc | HGVSp | HGVSp_Short |
| --- | --- | --- | --- |
| Sesn1 | c.1296N>T | p.Gln432His | p.Q432H |
| Ros1 | c.3094N>T | p.Ala1032Ser | p.A1032S |
| Arid5b | c.2807N>T | p.Gly936Val | p.G936V |
| Lats1 | c.1304N>A | p.Ser435Ter | p.S435* |
| Lats1 | c.2773N>T | p.Gly925Ter | p.G925* |
| Ikzf1 | c.1282N>A | p.Gln428Lys | p.Q428K |
| Trp53 | c.722N>A | p.Gly241Glu | p.G241E |
| Trp53 | c.787N>T | p.Gly263Ter | p.G263* |
| Pik3cg | c.2335N>A | p.Leu779Ile | p.L779I |
| Asxl2 | c.3187N>T | p.Gly1063Cys | p.G1063C |
| Prkd1 | c.1042N>T | p.Glu348Ter | p.E348* |
| Prkd1 | c.764N>T | p.Ser255Leu | p.S255L |
| Map3k1 | c.3722N>A | p.Ala1241Glu | p.A1241E |
| Map3k1 | c.1759N>C | p.Val587Leu | p.V587L |
| Hist1h1b | c.230_232delNNN | p.Asn77del | p.N77del |
| Jarid2 | c.604N>T | p.Ala202Ser | p.A202S |
| Syk | c.1715N>A | p.Gly572Glu | p.G572E |
| Tert | c.1561N>A | p.Ala521Thr | p.A521T |
| Arhgef28 | c.324N>A | p.Asp108Glu | p.D108E |
| Ubr5 | c.7839N>T | p.Gln2613His | p.Q2613H |
| Ubr5 | c.5299N>C | p.Ala1767Pro | p.A1767P |
| Kmt2d | c.223N>C | p.Gly75Arg | p.G75R |
| Ercc4 | c.2345N>A | p.Ser782Asn | p.S782N |
| Mapk1 | c.22N>T | p.Gly8Cys | p.G8C |
| Map3k13 | c.886N>C | p.Asp296His | p.D296H |
| Slx4 | c.139N>A | p.Pro47Thr | p.P47T |
| Axin1 | c.1753N>T | p.Gly585Cys | p.G585C |
| Dusp1 | c.1004N>A | p.Thr335Asn | p.T335N |
| Gm15821 | c.112N>A | p.Ser38Thr | p.S38T |
| Notch4 | c.2773N>A | p.Asp925Asn | p.D925N |
| Notch4 | c.3624N>T | p.Lys1208Asn | p.K1208N |
| Notch4 | c.4235N>T | p.Ala1412Val | p.A1412V |
| Mdc1 | c.4900N>T | p.Gly1634Cys | p.G1634C |
| Arid1b | c.5875N>T | p.Glu1959Ter | p.E1959* |
| Pdgfrb | c.1747N>T | p.Asp583Tyr | p.D583Y |
| Csf1r | c.292N>G | p.His98Asp | p.H98D |
| Csf1r | c.1223N>G | p.Pro408Arg | p.P408R |
| Setbp1 | c.2434N>T | p.Gly812Ter | p.G812* |
| Setbp1 | c.1377N>T | p.Lys459Asn | p.K459N |
| Prex2 | c.2756N>A | p.Pro919Gln | p.P919Q |
| Gm16432 | c.107N>A | p.Thr36Asn | p.T36N |
| Ercc5 | c.3433N>A | p.Val1145Ile | p.V1145I |
| Erbb4 | c.283N>T | p.Arg95Cys | p.R95C |
| Grem1 | c.206N>A | p.Pro69His | p.P69H |
| Bcl2l1 | c.363N>T | p.Gln121His | p.Q121H |
| Asxl1 | c.1811N>T | p.Gly604Val | p.G604V |
| Gnas | c.1811N>T | p.Arg604Leu | p.R604L |
| Dido1 | c.2995N>T | p.Ala999Ser | p.A999S |
| Notch1 | c.6073delN | p.Ala2025ArgfsTer4 | p.A2025Rfs*4 |
| Gm10801 | c.277N>A | p.Val93Ile | p.V93I |
| Tet2 | c.5224N>C | p.Glu1742Gln | p.E1742Q |
| Ntrk1 | c.1259N>C | p.Gly420Ala | p.G420A |
| Ntrk1 | c.925N>A | p.Gly309Arg | p.G309R |
| Lck | c.921N>T | p.Gln307His | p.Q307H |
| Ldlrap1 | c.626N>A | p.Gly209Glu | p.G209E |
| Runx1t1 | c.1305N>T | p.Arg435Ser | p.R435S |
| Mtor | c.6771N>T | p.Lys2257Asn | p.K2257N |
| Nbn | c.1278N>T | p.Lys426Asn | p.K426N |
| Lyn | c.194N>A | p.Gly65Asp | p.G65D |
| Lyn | c.733N>T | p.Glu245Ter | p.E245* |
| Lyn | c.1336N>C | p.Gly446Arg | p.G446R |
| Arid3c | c.654N>T | p.Arg218Ser | p.R218S |
| Arid3c | c.326N>A | p.Ala109Glu | p.A109E |
| Ptprd | c.2135N>T | p.Gly712Val | p.G712V |
| Ptprd | c.531N>G | p.Asn177Lys | p.N177K |
| Ptprd | c.191N>T | p.Trp64Leu | p.W64L |
| Ep400 | c.1401N>T | p.Lys467Asn | p.K467N |
| Ksr2 | c.285N>A | p.Trp95Ter | p.W95* |
| Dtx1 | c.589N>C | p.Gly197Arg | p.G197R |
| Setd1b | c.1366N>A | p.Gly456Ser | p.G456S |
| Setd1b | c.4525N>T | p.Val1509Phe | p.V1509F |
| Ncor2 | c.4483N>A | p.Ala1495Thr | p.A1495T |
| Cux1 | c.1857N>A | p.Ser619Arg | p.S619R |
| Brca2 | c.3970N>A | p.Pro1324Thr | p.P1324T |
| Brca2 | c.3971N>A | p.Pro1324Gln | p.P1324Q |
| Epha5 | c.2626N>C | p.Val876Leu | p.V876L |
| Pparg | c.374N>T | p.Arg125Met | p.R125M |
| Raf1 | c.817N>A | p.Asp273Asn | p.D273N |
| Ret | c.1939N>T | p.Leu647Phe | p.L647F |
| Pik3c2g | c.2594N>A | p.Arg865His | p.R865H |
| Mgam | c.2334N>T | p.Met778Ile | p.M778I |
| Mgam | c.4810N>C | p.Gly1604Arg | p.G1604R |
| Mgam | c.4990N>A | p.Asp1664Asn | p.D1664N |
| Gata2 | c.1304N>A | p.Gly435Glu | p.G435E |
| Smg1 | c.10303N>C | p.Ala3435Pro | p.A3435P |
| Smg1 | c.8949N>C | p.Met2983Ile | p.M2983I |
| Smg1 | c.8836N>T | p.Val2946Phe | p.V2946F |
| Smg1 | c.1156N>A | p.Val386Ile | p.V386I |
| Setd1a | c.4241_4242insGGGGGGGGGGGGG | p.Pro1415GlyfsTer15 | p.P1415Gfs*15 |
| Hras | c.35N>A | p.Gly12Glu | p.G12E |
| Cebpa | c.569N>T | p.Pro190Leu | p.P190L |
| Eed | c.1250N>A | p.Ser417Asn | p.S417N |
| Zfhx3 | c.2534N>C | p.His845Pro | p.H845P |
| Fgfr1 | c.963N>T | p.Lys321Asn | p.K321N |
| Pik3r2 | c.478N>T | p.Ser160Cys | p.S160C |
| Cyld | c.1495N>T | p.Val499Leu | p.V499L |
| Ephb1 | c.2563N>T | p.Gln855Ter | p.Q855* |
| Gm5611 | c.790N>T | p.Asp264Tyr | p.D264Y |
| Cbl | c.2332N>A | p.Asp778Asn | p.D778N |
| Cbl | c.70N>A | p.Leu24Met | p.L24M |
| Atm | c.6927N>A | p.Trp2309Ter | p.W2309* |
| Pgr | c.398N>A | p.Pro133Gln | p.P133Q |
| Pik3cb | c.2845N>T | p.Arg949Ter | p.R949* |
| Btk | c.949N>C | p.Val317Leu | p.V317L |
| Zrsr2 | c.1361N>A | p.Arg454His | p.R454H |
| Zrsr2 | c.736N>T | p.Asp246Tyr | p.D246Y |
| Kdm6a | c.31N>A | p.Ala11Thr | p.A11T |
| Amer1 | c.2238N>T | p.Arg746Ser | p.R746S |

**Supplementary Table 2**

**Table 2: List of antibodies used in this study.**

| Antibody Name | Catalogue no | Company | Application | Dilution |
| --- | --- | --- | --- | --- |
| AKT | 2964S | CST | WB | 1:1000 |
| pAKT (S473) | 4058S | CST | WB  IHC | 1:1000  1:200 |
| β-actin | 4967S | CST | WB | 1:1000 |
| Cleaved Caspase - 3 | C9664 | CST | IHC | 1:1000 |
| CD8 | 98941S | CST | IHC | 1:100 |
| CD45 | 70257S | CST | IHC | 1:100 |
| CD20 | 87887SF | CST | IHC | 1:100 |
| ERK1/2 (MAPK) | 4695S | CST | WB  IHC | 1:1000  1:100 |
| pERK1/2 (pMAPK) | #4695S | CST | WB  IHC | 1:1000 |
| Ki67 | Ab16667 | Abcam | IHC | 1:200 |
| PD-L1 | 238697 | Abcam | IHC | 1:200 |
| S6 | #2217 | CST | WB | 1:1000 |
| pS6 (240/244) | 5364S | CST | WB  IHC | 1:1000  1:300 |
| pS6 (235/236) | #4857S | CST | WB | 1:1000 |
